# Sensory Attenuation in the Auditory Modality as a Window Into Predictive Processing

**DOI:** 10.3389/fnhum.2021.704668

**Published:** 2021-11-05

**Authors:** Fabian Kiepe, Nils Kraus, Guido Hesselmann

**Affiliations:** Psychologische Hochschule Berlin (PHB), Berlin Psychological University, Berlin, Germany

**Keywords:** sensory attenuation, predictive processing, temporal control, temporal prediction, identity prediction, sense of agency (SoA)

## Abstract

Self-generated auditory input is perceived less loudly than the same sounds generated externally. The existence of this phenomenon, called Sensory Attenuation (SA), has been studied for decades and is often explained by motor-based forward models. Recent developments in the research of SA, however, challenge these models. We review the current state of knowledge regarding theoretical implications about the significance of Sensory Attenuation and its role in human behavior and functioning. Focusing on behavioral and electrophysiological results in the auditory domain, we provide an overview of the characteristics and limitations of existing SA paradigms and highlight the problem of isolating SA from other predictive mechanisms. Finally, we explore different hypotheses attempting to explain heterogeneous empirical findings, and the impact of the Predictive Coding Framework in this research area.

## Introduction

Sensory Attenuation (SA) describes the phenomenon that self-initiated sensory input is perceived with a lesser intensity than the same sensations generated externally (Hughes et al., 2013a; Pyasik et al., 2021). While many of us might have caught ourselves not noticing repeatedly clicking a ballpoint pen or tipping on the table, we perceive those sounds as noisy and intrusive when generated by another person (Klaffehn et al., 2019). The ability to differentiate one’s own action-related auditory signals from externally generated sounds not only aids movement coordination but can also inform us of potential threats (Myers et al., 2020).

For the scope of this review, we will focus on two major approaches that have been brought forward in order to explain SA following self-initiated action. Classical forward models of SA (Blakemore et al., 1999, 2002; Synofzik et al., 2008) propose that for self-initiated actions, the designated structures of the motor system are in constant exchange with each other, not only generating motor commands but also creating efference copies of these commands. These efference copies allow the brain to predict the resulting changes in sensory inputs caused by the intended behavior and subsequently subtract predicted from actual changes in sensory inputs, canceling out the sensory consequences of self-initiated behavior (Bays et al., 2008). The proposed main function of SA in these models is to anticipate and cancel the sensory effects of movement (Miall and Wolpert, 1996), thereby enabling the differentiation of self-initiated from externally caused changes in sensory inputs. Depending on the specific implementation of forward models, this information is subsequently used to make attributional judgments and facilitate a sense of agency (e.g., Synofzik et al., 2008). However, in recent years this view of SA has been challenged by applications of the broader theory of predictive processing.

## Predictive Processing

Predictive processing suggests that, not only for self-generated action but in general, we constantly make use of prior information in order to generate predictions about upcoming changes in sensory input in the form of a generative model (Friston et al., 2016). Possible deviations in actual sensory evidence from the predicted inputs (prediction errors) are used to update the current model and inform predictions in subsequent processing. This continuous Bayesian updating scheme enables inference of hidden states causing changes in the environment by comparing changes in predicted with actually detected sensory inputs, providing the basis for intero- as well as exteroception (Seth et al., 2012). During this process, the brain is constantly aiming at maximizing model evidence (i.e., to increase the utility of the predictive model) by minimizing prediction error and surprise. Further, the principle of active inference within predictive processing states that motor behavior plays an important role in achieving this (Parr et al., 2021). Self-initiated action herein serves the purpose of altering one’s physical surroundings so that received sensory inputs match the predicted ones, thereby minimizing prediction errors. By informing involved systems about the desired state, predictions are the driving force for resulting self-initiated movements. Beforehand, however, there is a crucial time interval wherein the predicted outcome and the actual sensory input are yet to match. During this period, the signals stemming from self-initiated behavior are attenuated, signaling that these stimuli stem from self-actions (Aru, 2019). SA specifically would arise from lowering the precision of anticipated sensory events, being equivalent to drawing away attentional resources from these inputs (Brown et al., 2013).

The differences in the two portrayed explanatory frameworks may seem negligible when trying to explain everyday phenomena of sensory attenuation, but they bear important implications for the explanation of partially conflicting results in scientific research on SA. It is important to note that the two models make different assumptions over the function of SA in auditory perception. In forward models, SA enables the differentiation between externally and internally caused sensory signals. Information from motor regions in the brain is, therefore, a necessary condition for SA in forward models, since all self-generated auditory signals will be caused by motor activity. However, in predictive processing, motor information is only the expected precision (i.e., predictability) of a stimulus rendering it valuable in further processing (Friston, 2013). Therefore, predictive processing would imply attenuation of all anticipated sensory stimuli, independent of whether a self-initiated motor response was the perceived cause. Note, however, that an internally planned motor response is an especially reliable source of information rendering its anticipated auditory consequences unusually precise, thus facilitating SA. It follows that SA would be present in all expected stimuli but especially pronounced in expected self-generated ones.

In contrast to forward models, predictive coding does not conceive SA as a result of reafference cancellation. Rather, attenuation of expected signals is a logical conclusion from the imperative to minimize surprise and allocate attention and processing resources to unexpected stimuli since those are most effective in model updating. Importantly, this framework stresses the usefulness of predictive information on self-generated movements in creating a sense of agency (Kahl and Kopp, 2018). It does, however, not imply that sensory attenuation would necessarily follow from this. Looking at forward models, on the other hand, it is not apparent why self-generated signals should be attenuated, rather than amplified or distorted in any other fashion, since the predictive signal mainly serves the function of enabling differentiation between self and other. SA alone is likely insufficient in providing this information since an attenuated self-generated stimulus is subjectively hardly distinguishable from the same externally produced stimulus presented with less intensity (Burin et al., 2017). Alternatively, in order to differentiate between self and other generated stimuli, the perceptual systems could rely on a sense of *otherness*, as is present when hearing ones’ own voice on tape, rather than attenuated processing.

In what follows, we will try to further disentangle the specific implications of both explanatory approaches and identify the strengths and weaknesses by comparing their potential to explain several recent empirical findings. Note that reasons for contradicting results might also stem from the wide variety of methods used, as well as from the lack of a single coherent theoretical framework.

## Studying Sensory Attenuation in The Auditory Domain

**Figure 1:**
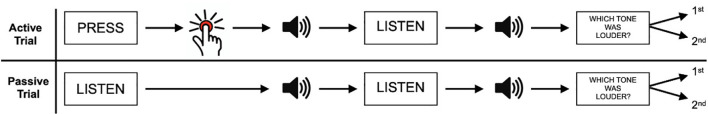
A typical experimental setup examining sensory attenuation in the auditory paradigm. In active trials, participants have to self-initiate a sound (e.g., through a button press) and compare its volume to an externally generated sound. In passive trials, both sounds are generated externally. Adapted from Reznik et al. (2015). Copyright (2015) by Reznik et al.

Typical setups in behavioral SA research consist of a two-phase comparison task that either contains an externally triggered stimulus or a self-initiated stimulus (Figure 1). This stimulus is then compared to a consecutive second, often identical, stimulus. For the auditory domain, the participants usually produce a sound by keypress. Consecutively, the identical stimulus reappears without the participant’s action, i.e., generated by a computer or another person. Thereafter, participants must compare or rate the volume of self-initiated vs. externally generated stimuli (Reznik et al., 2015). Participants then typically rate the self-initiated sound significantly lower in volume, compared to the externally generated signal (Reznik et al., 2015; Myers et al., 2020). Attenuation effects are not only studied using subjective measurements of perception but also in neuronal recordings of early stimulus-evoked brain activity. Studies using Electroencephalogram (EEG) or magnetoencephalogram (MEG) for example do not have to rely upon delayed behavioral responses reporting subjective attenuation effects that potentially are subject to post perceptual judgment biases, but in principle offer real time measures of auditory perception. They also provide further benefit in that they offer a measure of SA in no-report paradigms, in which participants are asked to passively perceive a (potentially cued) sound-isolating SA from effects of motor planning and execution. In EEG studies that nevertheless do involve a self-initiated action, ERPs are typically corrected for motor behavior components. Such studies have revealed a reduction in amplitude of auditory event-related potentials (ERP; N1 and P2) when initiating endogenous sounds, such as speaking or blowing air, compared to externally generated auditory stimuli (Ford et al., 2007; Mifsud and Whitford, 2017).

## Confounds of Temporality

There are mainly two temporal mechanisms influencing the effect of SA: temporal predictability and temporal control. Temporal predictability describes the ability to predict the point in time at which a sensory event will occur. Temporal control, on the other hand, defines the ability to control the time of the stimulus onset through one’s own behavior (Hughes et al., 2013a). When contrasting different explanatory models for SA, empirically disentangling the respective contributions of temporal predictability and control to SA becomes an especially important tool. Predictive processing considers the predictability of a stimulus central to its potential to elicit attenuated processing, and while direct control over stimulus appearance certainly should enhance predictability, it is not conceived as a mandatory requirement for SA. Forwards models, however, posit self-initiated motor behavior as a necessary requirement for SA, while making no assumptions over the role of predictability alone.

One effective tool to manipulate temporal predictability is delaying the onset of the stimulus. Several studies have shown attenuated N1 components despite (randomized) stimulus onset delays of up to 1,000 ms, suggesting that SA is not dependent on temporal predictability alone (Lange, 2011; van Elk et al., 2014; Klaffehn et al., 2019).

Recent studies tried to further disentangle the individual contributions of temporal control and temporal predictability to SA. Kaiser and Schütz-Bosbach (2018) demonstrated that significant attenuation of N1 to an auditory stimulus takes place when it is highly predictable but not self-generated and only passively perceived. They further show that N1 is not attenuated but elevated for trials in which participants were asked to press a button in reaction to a cue (thereby self-initiating the tone) compared to when they were asked to passively perceive the same cue. This not only stresses the relative importance of predictability compared to self-initiation for SA but also illustrates the shortcomings of forward models to explain SA when no motor behavior takes place. However, Klaffehn et al. (2019) found only a small influence of temporal predictability (manipulated by a 750 ms progress bar leading up to the stimulus) on P2 but not N1 amplitudes. Looking only at self-initiated actions, N1 showed strong attenuation effects to tones that were played immediately compared to when they were temporally delayed (750 ms) and preceded by a progress bar. Moreover, by implementing cued trials (visual stimuli indicating the timing of auditory stimulus onset) and uncued trials (random visual stimuli unrelated to auditory stimulus onset or action), Harrison et al. (2021) could isolate the effects of temporal predictability and temporal control and found that both mechanisms do independently contribute to attenuation. Note that in this study, temporal control had the usual facilitating effect on SA in the P2, but looking at the N1 effect patterns were reversed with higher temporal control leading to reduced attenuation of the ERP. The authors summarize that taken together, both factors (temporal predictability and temporal control) do not sufficiently explain the observed overall effect size of SA. These findings thus further highlight the rather strong relative importance of self-initiation on SA, potentially surpassing its contribution to the temporal predictability of a stimulus alone. Establishment and replication of the finding that self-initiation contributes more to SA than facilitating the (temporal) predictability of a stimulus would question the inherent logic of predictive processing models.

## Confounds of Identity Prediction

Identity prediction describes the ability to predict the identity of the stimulus, based on self-initiated behavior (motor-based identity prediction) or other cues (non-motor-based identity prediction; Hughes et al., 2013a). Consistent with motor-based and prediction-based models, several studies show that motor identity prediction regulates SA (Hughes et al., 2013a). As for factors of temporal predictability, the question of whether and how non-motor-based identity prediction significantly contributes to SA can help us evaluate the utility of forward models. Since in those models prediction of subsequent changes of sensory inputs is solely based on motor-based efference copies, non-motor-based identity prediction should not contribute to SA. In predictive processing theories, not only self-generated action but also external information gathered across all sensory domains contributes to the prediction of subsequent sensory inputs, rendering identity prediction a useful mechanism contributing to SA (Talsma, 2015).

By studying the effect of self-initiated action on SA in trials of varying stimulus qualities, several experiments show significantly enhanced SA for motor identity prediction. Hughes et al. (2013b), for example, taught participants specific action-sound combinations and found significantly stronger N1 attenuation for stimuli that were coherent with previously learned contingencies, compared to non-coherent action-sound combinations. Baess et al. (2008) compared trials where the pitch of self-initiated sounds was constant (1,000 Hz), and thus predictable, with trials where the pitch was randomized (400–1,990 Hz), and thus unpredictable for participants. When the identity of the sound could be predicted, SA was significantly increased, compared to when it was not. The effect could further be isolated from self-generation of the stimulus in a passive listening paradigm, where identity could only be predicted on the basis of the previous tones (non-motor-based identity prediction; Lange, 2009). This poses a challenge to classical (or, auxiliary) forward models of SA, according to which predictions are solely based on efference copies of motor commands (Pickering and Clark, 2014). According to alternative specifications of forward models, however, SA is not simply a reflection of the efference copy. Specifically, the prediction of sensory outcomes in these models can be based on efference copies as well as on learned sensory associations (Pickering and Clark, 2014).

Dogge et al. (2019b), however, could only find a weak influence of identity prediction on SA, and no difference in influence between motor and non-motor identity prediction in forced choice tasks measuring different ERPs. Taken together, it seems that identity prediction, in general, can enhance SA, but cannot solely account for it. Further, motor-based identity predictions alone cannot account for the majority of the SA results (Horváth, 2015; Dogge et al., 2019b).

## Attention Vs. Prediction

If SA relies entirely on motor-based prediction, attention towards a specific stimulus, which cannot be predicted, should not alter the overall effect of SA (Wiese, 2016). Indeed, several studies investigating attention-based explanations of SA suggest that attention effects may not be sufficient in explaining attenuation of self-generated actions and that both effects might be additive rather than intertwined with each other (Saupe et al., 2013). No significant differences in auditory ERP attenuation were found if attention was allocated towards non-auditory sensory input, motor behavior, or auditory stimuli (Timm et al., 2013; Neszmélyi and Horváth, 2021). However, other studies could show that attention increases sensory processing in SA paradigms, even outweighing the effects of SA in certain cases. In a sound detection task, Cao and Gross (2015) asked participants to attend to a specific target sound. Although there were no differences between the presented tones with regard to temporal predictability, attention towards a specific sound led to less SA, compared to the other tones. It is, however, difficult to disentangle the respective contributions of attention and prediction to SA, since attention generally should facilitate predictive abilities (Alink and Blank, 2021). While both mechanisms, attention and prediction, are thought to aid perception, their relationship is still up to debate (Schröger et al., 2015).

While prediction has been shown to decrease N1 and P1 components in auditory perception thus attenuating early auditory perception, attention was found to increase the perception of sensory inputs (Lange, 2013; Schröger et al., 2015). The heterogeneity of SA results, and the issues of temporality and identity prediction, might stem from difficulties in isolating these opposing mechanisms (Lange, 2013). But how do prediction and attention interact? Several studies show that attention to stimuli often results in elevated ERPs (N1 and P2) to those stimuli. However, if participants are instructed to execute a certain movement (e.g., a keypress), attention might be mainly allocated towards that action, drawing away attentional resources from subsequent perceptual processing. In auditory tasks, in which participants are instructed to solely listen and not to move, attention can be distributed fully towards the stimulus (Horváth, 2015). The heterogeneity of SA study results might thus stem from differences in attention orienting, depending on the study’s design.

In a series of recent experiments, participants were instructed to press a button in a virtual environment during an auditory forced choice task. This allowed the researchers to detach tactile feedback from motor behavior (Fritz et al., 2021). Results suggest that SA for auditory stimuli only occurs if attention is oriented towards a different stimulus (e.g., tactile input deriving from the preceding movement), and away from the auditory modality. In a sound detection task by Reznik et al. (2015), the influence of sound intensity on SA was examined. Their study showed that for self-initiated tones with high intensity, the volume was attenuated. However, for self-initiated tones with low intensity, the volume was enhanced, suggesting that, for sounds with near-threshold volume, attention may be drawn towards these stimuli. Similar phenomena can also be observed in studies examining learned behavior. If certain action-stimulus combinations are learned, its perception of the stimulus is easier to predict. Therefore, attention can be oriented elsewhere. In an auditory forced choice task measuring EEG, Dogge et al. (2019b) could only observe attenuation of self-triggered stimuli if the connection between action and effect was trained properly beforehand, during a sufficient acquisition phase.

Attenuating expected stimuli at least partly dependent on the altered allocation of attention is also hypothesized in some predictive processing approaches to SA (Chennu et al., 2016; Wiese, 2016; Dogge et al., 2019a). According to predictive coding, attention is conceived as synaptic gain control, thereby regulating the precision of prediction errors at all levels of cortical processing (Chennu et al., 2016). Prediction on the other hand is thought of as top-down information flow including specific contents as well as precision, mediating the response of lower processing levels to incoming sensory evidence. These two processes would therefore be naturally interdependent, considering that prediction can influence synaptic gain at lower processing levels to specific inputs. Note, however, that additional mechanisms have been brought forward describing how prediction could lead to SA, other than modulating attention (Schröger et al., 2015; Alink and Blank, 2021).

## Sense of Agency

Another mechanism possibly influencing SA is the Sense of Agency (SoA). It describes the individual’s awareness of control over self-initiated actions (Jeannerod, 2003). The efficient differentiation between internally and externally generated changes of sensory inputs might be a crucial component for the development of a coherent SoA. With disturbed agency being one of the explanations for schizophrenia symptoms, neurophysiological studies compared attenuation effects between healthy individuals and patients with diagnosed schizophrenia. They found reduced N1 attenuation for self-initiated behavior in schizophrenic patients (Ford and Mathalon, 2012).

A widely accepted connection between SA and SoA, however, has not been established yet. While SA appears to take place in low-level processing and in the first 200 ms after stimulus onset, SoA requires a higher and potentially later level of processing (Dewey and Knoblich, 2014; Wolpe and Rowe, 2014; Wen et al., 2019). Moreover, differences in study results might be explained by the difficulty of measuring SoA (Haggard and Chambon, 2012).

In a study by Timm et al. (2016), SoA was manipulated by altering learned delays for certain action-sound combinations. During an acquisition phase, participants learned that, after button press, the sound succeeds following a fixed delay (e.g., 200 ms). The test phase included trials with shortened delays (e.g., 0 ms), causing participants to perceive that the sound preceded their action, resulting in a lack of agency. Results showed that N1 attenuation for self-initiated sounds is not dependent on agency judgments. However, P2 attenuation appears to correlate with participants’ SoA. Other observations underline the difficulty of placing SoA into motor-based forward models. Weiss et al. (2011) compared perceived subjective loudness of self- vs. other-initiated tones, and subdivided the trials into “interactive” and “individual” trials: interactive (1. self-generated, but other-initiated; 2. other-generated, but self-initiated) and individual (3. self-initiated and generated; 4. other-initiated and generated). During the interactive trials, the participants interacted with the experimenter (through taps on the shoulder) to trigger the stimuli. During the individual trials, there was no interaction between the participants and the experimenter. Significant differences in SA were found between all conditions including SoA (self-generated, but other initiated; other-generated, but self-initiated; self-initiated and generated) and the condition not containing SoA (other-initiated and generated), suggesting that having an SoA over specific actions affects perception. Interestingly, attenuation was strongest in the condition in which the button press was self-generated but other-initiated. This suggests that while SoA can influence SA, it might not be the only mechanism responsible for attenuation effects. Rather, it appears that an additional source informing us about incoming information (e.g., another person tapping us on our shoulder) helps us to successfully predict sensory input (Weiss et al., 2011).

Other studies showed that, although sounds were always generated by the participants themselves, there were differences in SA depending on their belief in agency. Desantis et al. (2012), for example, could show that framing participants into believing that another person triggered the stimuli had an influence on SA, although the sounds were always triggered by the participants themselves. Participants rated the volume of sounds they believed to be self-initiated as lower than the sounds they believed to be externally generated. Borhani et al. (2017) let the participants decide in which pitch range (low or high) the sound stimulus should appear, and showed that the belief of free choice alone can alter SA. These studies underpin the effects of SoA on SA, which are difficult to explain by motor-based forward models. If the motor command, and thus its efference copy, stays the same throughout all trials, there should not be differences in SA based on differences in SoA alone, according to forward models. While motor-based forward models mainly suggest SoA to be formed after stimulus onset, several studies could show that SoA can be influenced by mechanisms prior to action outcomes, like motor intention, the belief of agency, and free choice over designated action effects (Haggard and Chambon, 2012). As stated above, predictive processing additionally emphasizes the importance of predictive information for creating SoA (Kahl and Kopp, 2018). In line with the studies discussed above, this framework also omits the necessity that SA develops as a consequence of SoA, or* vice versa* (Burin et al., 2017). Rather, attenuation of expected signals may be the result of the imperative to reduce surprise and therefore reduced allocation of attention to predicted stimuli.

## Summary

Focusing on auditory studies, this review summarized recent developments in SA research and discussed the strengths and weaknesses of two major theoretical frameworks, forward models and predictive processing. Results of current studies examining the confounding effects of temporality indicate that while temporal predictability and control indeed influence attenuation effects, other mechanisms must be included to explain SA (Kaiser and Schütz-Bosbach, 2018; Harrison et al., 2021). Studies investigating the role of identity prediction could show SA based on learned associations rather than motor- vs. externally-generated behavior (Schröger et al., 2015; Dogge et al., 2019b). These results suggest SA to be a result of attention orienting based on the prediction that is not necessarily dependent upon motor behavior (Schröger et al., 2015; Chennu et al., 2016; Wiese, 2016; Dogge et al., 2019a). By manipulating attention orientation, multiple studies showed that, while self-initiated motor behavior is a reliable predictor, it does not necessarily lead to SA. Similarly, several studies observed the importance of cues prior to and after stimulus onset for the sense of agency and stated its impact on, but not its necessity for the development of SA.

Classical forward models depend on motor commands to predict and subsequently attenuate sensory inputs, thereby giving the agent the possibility to differentiate between self- and other generated stimuli, and thus facilitating a sense of agency. These models cannot account for several phenomena of SA that were observed independent from motor behavior, the strong role of attention in SA, as well as the influence of agency beliefs on SA prior to stimulus onset. Predictive processing, on the other hand, states that we constantly make use of prior information, either self- or externally-generated, in order to create predictions about upcoming changes in sensory input in the form of a generative model (Friston et al., 2016). In this framework, only the predictability of a stimulus should determine its potential to elicit SA. This partially contradicts a consistent finding throughout the literature, namely that even when a stimulus is reliably predicted by external cues, self-generation of a motor behavior does still individually contribute a significant part to SA effects. Although self-initiated action serves as a reliable predictor for generating inferences, further research is needed to elucidate its central role in SA, leaving room for new explanatory hybrid models (Dogge et al., 2019a). Such models combine the existence of an efference-copy-based forward model with a global predictive mechanism. The forward model in this approach is still based only on motor action, potentially providing more efficient processing of contingencies that are especially reliable since they are self-initiated as well as deeply learned and reinforced over a time course of years, such as the production and perception of one’s own voice. The global predictive mechanism on the other hand would provide a more flexible and adaptive tool in order to anticipate newly learned contingencies in an ever-changing environment. Further studies testing the assumption of differential processing of motor and non-motor-based predictive information is certainly needed to elucidate the utility of such hybrid models.

## Author Contributions

FK, NK, and GH wrote the manuscript. All authors contributed to the article and approved the submitted version.

## Conflict of Interest

The authors declare that the research was conducted in the absence of any commercial or financial relationships that could be construed as a potential conflict of interest.

## Publisher’s Note

All claims expressed in this article are solely those of the authors and do not necessarily represent those of their affiliated organizations, or those of the publisher, the editors and the reviewers. Any product that may be evaluated in this article, or claim that may be made by its manufacturer, is not guaranteed or endorsed by the publisher.
